# SiDIVS: Simple Detection of Inductive Vehicle Signatures with a Multiplex Resonant Sensor

**DOI:** 10.3390/s16081309

**Published:** 2016-08-17

**Authors:** José J. Lamas-Seco, Paula M. Castro, Adriana Dapena, Francisco J. Vazquez-Araujo

**Affiliations:** Department of Electronics and Systems, University of A Coruña, 15071 A Coruña, Spain; lamas@udc.es (J.J.L.-S.); adriana@udc.es (A.D.); fjvazquez@udc.es (F.J.V.-A.)

**Keywords:** data acquisition, inductive loop detector, instrumentation and measurements, intelligent transportation systems, multiplex systems, vehicle inductive signature

## Abstract

This work provides a system capable of obtaining simultaneous inductive signatures of vehicles traveling on a roadway with minimal cost. Based on *Time-Division Multiplexing* (TDM) with multiple oscillators, one for each inductive loop, the proposed system detects the presence of vehicles by means of a shift in the oscillation period of the selected loop and registers the signature of the detected vehicles by measuring the duration of a fixed number of oscillator pulses. In order to test the system in an actual environment, we implement a prototype that we denote as SiDIVS (Simple Detection of Inductive Vehicle Signatures) and acquire different vehicle inductive signatures under real scenarios. We also test the robustness of the detector by simulating the effect of noise on the signature acquisition.

## 1. Introduction

One of the most important aspects of *Intelligent Transportation Systems* (ITS) is vehicle traffic monitoring, essentially those applications whose aim is to count the number of vehicles on a roadway or to know their speed, occupancy, or structural characteristics like density and type.

The sensors used for these applications can be classified, according to their location in the road, as in-roadway and over-roadway. In general, in-roadway sensors may have problems of installation and maintenance, whereas over-roadway sensors suffer from blocking of *Line-Of-Sight* (LOS) by weather conditions or obstacles. However, the most frequently-used classification of sensors is done according to the existence or not of an external energy source. Thus, we can talk of active and passive sensors [1,2,3], respectively. Passive sensors measure reflected radiation that has been emitted from the surrounding environmental elements. Some examples of passive sensors are image processing, acoustic, seismic or magnetic sensors. Active sensors require their own source of excitation and measure the reflected energy. Radar, laser, infrared, ultrasound and emerging technologies-based sensors are some examples that lie in this category.

Since their introduction in the 1960s, the active sensors known as *Inductive Loop Detectors* (ILD) are the more commonly used sensors in traffic management systems. This type of sensor has the advantages of being a highly developed technology; of a simple operation, unaffected by environmental conditions; and of a low installation cost. Although they have the problem of complex replacement in case of breakage, with the inevitable interruption of traffic, and need regular calibration, their flexible design can adapt to a large variety of applications, providing better accuracy in traffic classification than other commonly used techniques [1]. Thus, ILDs have been widely used for tasks such as vehicle classification [4,5,6,7,8], vehicle re-identification [9,10,11,12], and speed estimation with a single loop [13,14].

Several works set the basis for the theoretical study of ILDs. In [15,16], an approximate model for an ILD is shown, and an equivalent circuit model is detailed in [16]. A detailed study on the sensitivity of an inductive loop and the response time to changes in inductance of different digital detectors is shown in [1]. The work in [17] addressed the sensitivity problems in dual loops and proposed software and hardware implementation solutions to identify and correct them. In [18], three-dimensional maps illustrating the sensitivity of various inductive loops located under the asphalt surface of roads are obtained.

These works have led to different implementations of ILDs. In the US4680717A patent [19], a multiplex system for vehicular traffic detection with a single oscillator is presented. A multiple vehicle detection system incorporating low-cost oscillators and an algorithm to calibrate the device following changes in environmental conditions is proposed in [20]. However, although both works allow the detection of the presence of vehicles, neither of them register their inductive signatures. In [21], a system to detect multiple vehicles is also developed, but it requires multichannel acquisition of analogue signals, which makes it overly complex. Furthermore, due to the function not being fully multiplexed and to the use of the same frequencies in near loops, such development causes significant interferences between channels (also known as *crosstalk*). The work in [22] also presents a very complex hardware with a detector that obtains inductive vehicle signatures by measuring changes in coil impedance, separating its real part (R) from its imaginary part (X). It uses an analogue hardware, which integrates a self-balanced bridge when no vehicles are present, and two synchronous demodulators for obtaining the R and X signatures corresponding to each vehicle.

The US691182982 patent [23] presents equipment to produce a vehicle inductive signature by means of a change in inductance induced in the vehicle-loop when this vehicle passes over the road loop. Although this amplitude detector allows multiple vehicle detection, it requires complex and expensive analogue processing and signal acquisition circuits.

Taking into account the drawbacks identified in the aforementioned works, in this paper, we propose a multiplex system for the Simple Detection of Inductive Vehicle Signatures (SiDIVS). Our proposal implies a fully multiplexed system that avoids the interference between loops (also called *inter-loop interference*) thanks to a very simple and almost fully automatic digital measurement process. Therefore, it does not require the use of complex and expensive analogue processing circuits or of analogue signal acquisition methods.

The paper is organized as follows. Section 2 presents a brief theoretical study of ILDs. The models used to study the impact of noise on amplitude and resonant detectors are presented in Section 3. Section 4 shows the practical implementation of the SiDIVS prototype using a multiplex detector with eight channels. Section 5 explains the experimental measurements performed to evaluate the performance of such digital detectors with the inductive signatures captured by our prototype. Section 6 presents an analysis of the impact of the noise on these digital detectors. In addition, some examples of real inductive signatures collected from different vehicles are included. Finally, Section 7 is devoted to the conclusions.

## 2. Inductive Loop Detectors

**Figure 1:**
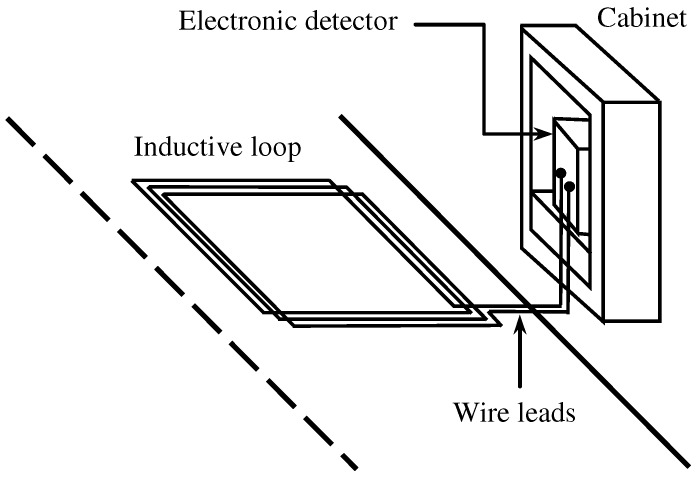
Elements of an inductive loop detector.

Figure 1 depicts the elements of an ILD. It consists of one or more coils with one or more turns (usually three to five) embedded in the road pavement; isolated cables for the connection from the coils to the control cabinet; and the electronic equipment (i.e., the detector) inside the cabinet.

The sensitivity *S* of an inductive loop is a measurement of its ability to detect small changes in inductance and is defined as the ratio between the change in inductance due to passing traffic and the initial inductance (i.e., the inductance when a vehicle is not present). Thus, we can express the sensitivity as

(1) $$S=\frac{\Delta L}{L_{nv}}=\frac{L_{nv}-L_{v}}{L_{nv}},$$

where $L_{nv}$ is the initial inductance when no vehicle is present, and $L_{v}$ is the inductance when a vehicle is present, so that the change in inductance is defined as $\Delta L=L_{nv}-L_{v}$.

Modern inductive detectors of vehicle presence are digital because they provide more reliable, accurate, and precise measurements than analogue detectors. Although there exist ILDs measuring other types of variations in the coil, like impedance [22], currently, the majority of ILDs indirectly measure variations in inductance as indicated in Equation (1). These variations are caused by the presence of a vehicle in the detection area of the inductive loop, which produces a decrease of inductance. Two methods are used to measure such variations: one based on measuring the frequency or period changes of an oscillator resonant circuit, and one based on measuring the voltage amplitude changes of an RLC circuit operating at a fixed frequency, known as *resonant ILDs* and *amplitude ILDs*, respectively. We briefly describe both ILD types in the following subsections.

### 2.1. Resonant ILDs

Resonant ILDs are based on the measurement of changes of oscillation frequency or period. The oscillator frequency is controlled by a parallel resonant circuit, also called *tank circuit*, which is constituted by a non-ideal loop with inductance *L* in serial with a resistor *R*, and this serial set connected in parallel with a capacitance *C* placed in the detector. The complex impedance of this circuit is given by:

(2) $$Z\left(jw\right)=\frac{R\left(1-w^{2}LC\right)+w^{2}RLC+j\left(wL\left(1-w^{2}LC\right)-wR^{2}C\right)}{{\left(1-w^{2}LC\right)}^{2}-w^{2}R^{2}C^{2}}.$$

Since for a resonant circuit it verifies Im[Z(jw)]=0, we have that $w_{0}L\left(1-w_{0}^{2}LC\right)-w_{0}R^{2}C=0$, which gives us the resonant angular frequency $w_{0}$

(3) $$w_{0}=\sqrt{\frac{L-R^{2}C}{CL^{2}}}.$$

Since our detector has R≈1Ω, L≥50μH, and C≤100nF, it verifies $L\gg R^{2}C$, and we can approximate $w_{0}$ by:

(4) $$w_{0}≅\sqrt{\frac{L}{CL^{2}}}=\frac{1}{\sqrt{LC}},$$

so that the oscillation frequency $f_{0}$ is given by:

(5) $$f_{0}=\frac{w_{0}}{2\pi }≅\frac{1}{2\pi \sqrt{LC}}.$$

It is important to note that the oscillation frequency depends on the inductance as $f_{0}=kL^{-1/2}$, with $k={\left(2\pi \sqrt{C}\right)}^{-1}$, and the frequency change is given by $\Delta f=f_{v}-f_{nv}$, $f_{v}$ being the oscillation frequency with vehicle presence, and $f_{nv}$ the oscillation frequency without its presence. Thus, we have:

(6) $$f_{v}=kL_{v}^{-\frac{1}{2}}=k{\left(L_{nv}-\Delta L\right)}^{-\frac{1}{2}}=kL_{nv}^{-\frac{1}{2}}{\left(1-\frac{\Delta L}{L_{nv}}\right)}^{-\frac{1}{2}}=f_{nv}{\left(1-\frac{\Delta L}{L_{nv}}\right)}^{-\frac{1}{2}},$$

with $f_{nv}=kL_{nv}^{-1/2}$, and

(7) $$\frac{\Delta f}{f_{nv}}=\frac{f_{v}-f_{nv}}{f_{nv}}=\frac{f_{v}}{f_{nv}}-1=\frac{1}{\sqrt{1-\frac{\Delta L}{L_{nv}}}}-1.$$

Since $\Delta L/L_{nv}$ is very small, $\Delta f/f_{nv}$ can be approximated by the first two terms of the Taylor series, i.e.,

(8) $$\frac{\Delta f}{f_{nv}}\approx \frac{1}{2}\frac{\Delta L}{L_{nv}}=\frac{1}{2}S,$$

where *S* is the sensitivity of the inductive loop (see Equation (1)). This sensitivity can thus be approximated by:

(9) $$S=\frac{\Delta L}{L_{nv}}\approx 2\frac{\Delta f}{f_{nv}}=2\frac{f_{v}-f_{nv}}{f_{nv}}=2\frac{T_{nv}-T_{v}}{T_{v}}=2\frac{\Delta T}{T_{v}},$$

where $T_{v}=1/f_{v}$ is the period of oscillation if a vehicle is over the coil, and $T_{nv}=1/f_{nv}$ denotes the period of oscillation otherwise. Experimental results have shown that the loop sensitivity *S* is extremely repeatable for fixed sizes and geometries of both the loop and the vehicle and for a fixed distance between them, as can be verified from Equations (1) and (9).

Detectors whose operation is based on period changes (i.e., based on period shifts ΔT), known as *type III* or *type IV*, in which period shifts or relative period shifts are measured, respectively, present a measurement time that is short enough for their use in applications of inductive signature capturing, although the characteristics of the oscillation loop have influence on the threshold sensitivity.

*Type III* detectors based on period shift use a reference clock signal whose frequency is of several MHz, typically between 20 and 1000 times greater than the oscillation frequency of the inductive loop we are interested in measuring. The period of the oscillation signal is calculated as the number of cycles *N* of the reference clock signal in *m* cycles of the oscillation signal. When a vehicle stops or passes over the loop, the oscillation frequency increases; thus, the period (and thus the number of cycles *N*) decreases. The counter of clock signal periods without vehicles involved is given by:

(10) $$N_{nv}=\frac{mT_{nv}}{T_{r}},$$

where $T_{r}$ is the period of the reference clock signal. On the other hand, with the presence of a vehicle over the loop, the number of cycles is calculated as:

(11) $$N_{v}=\frac{mT_{v}}{T_{r}}.$$

The shift ΔN can then be calculated as the difference between the values given by Equations (10) and (11) as:

(12) $$\Delta N=N_{nv}-N_{v}=\frac{m}{T_{r}}\left(T_{nv}-T_{v}\right)=m\frac{\Delta T}{T_{r}}.$$

Equating this value to the minimum detection threshold $N_{t}$ gives us:

(13) $$\Delta N=m\frac{\Delta T}{T_{r}}=N_{t}\;\to \;\Delta T=\frac{N_{t}T_{r}}{m},$$

so from Equation (9), we obtain the threshold sensitivity $S_{t}$ as:

(14) $$S_{t}≅2\frac{\Delta T}{T_{v}}=2\frac{N_{t}T_{r}}{mT_{v}}=2\frac{N_{t}}{N_{v}}.$$

From this equation, it can be seen that, for *type III* detectors, there is a loss in threshold sensitivity for high oscillation frequencies, although this loss can be easily reduced by increasing the frequency $f_{r}$ corresponding to the reference clock signal.

Most digital detectors can operate with four or more loops. The problem of crosstalk for resonant ILDs is solved by separating the loops, in our proposal up to eight directly connected to the detector, using *Time-Division Multiplexing* (TDM) [19,21]. The multiplexing could be extended to the detectors in the surrounding area by using synchronizing signals generated by one of them, which would work as the master, thus sequencing the time multiplexing of all the detectors. However, this has the disadvantage of reducing the sampling frequency of the obtained vehicle inductive signatures. These multiplexed models sequentially feed and analyse the channels more than 100 times per second using period shift detectors which, as mentioned before, are fast enough to allow these scanning rates.

**Figure 2:**
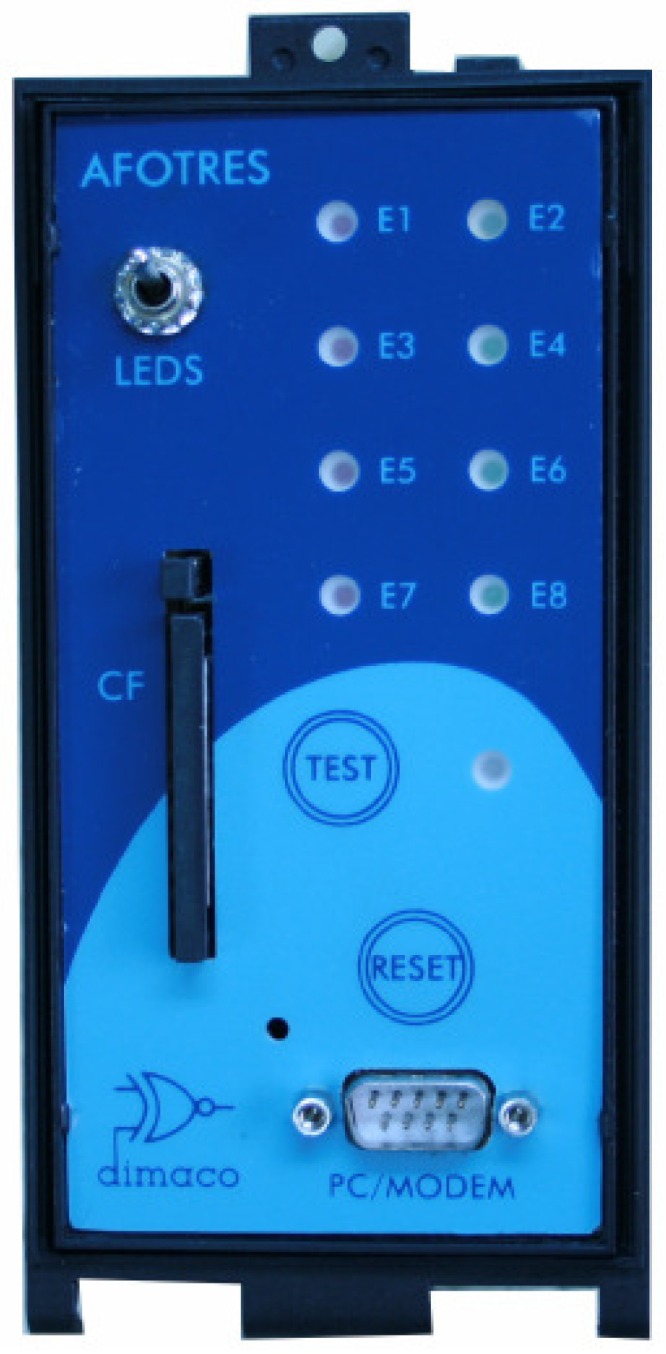
Eight-loop vehicle inductive detector (Afotres–Dimaco).

Our practical implementation is based on a *type III* detector using TDM, as we will detail in Section 4. We can see a real vehicle inductive detector of eight loops in Figure 2.

The oscillation frequency with a negligible resistance *R* is given by:

(15) $$f\left(t\right)=\frac{1}{T(t)}=\frac{1}{2\pi \sqrt{L(t)C}},$$

where the equivalent inductance L(t) is $L_{nv}$, with no presence of vehicle, or $L_{v}\left(t\right)$, with vehicle. For a resonant oscillator, when a vehicle is passing over the loop, both the loop inductance and the oscillation period decrease.

The signal at the oscillator output is expressed as:

(16) $$x\left(t\right)=A\sin\left(2\pi f(t)\right).$$

Then, the vehicle inductive signature is the period shift, expressed as $\Delta T=T_{nv}-T_{v}\geq \;0$. Then, the shift in the oscillation period (which gives us the inductive signature) is determined as follows:

(17) $$\Delta T\left(t\right)=2\pi \left(\sqrt{L_{nv}C}-\sqrt{L(t)C}\right).$$

### 2.2. Amplitude ILDs

Amplitude ILDs are based on the measurement of changes in voltage amplitude of an RLC circuit to which a fixed frequency signal is applied [21,23,24]. The RLC circuit is formed by the loop inductance *L* and both the resistance *R* and the capacity *C* in the detector, and it is connected to the sinusoidal voltage generator $V_{g}$ operating at a fixed frequency f=w/2π. The amplitude of the output voltage $V_{0}$ changes with the value of the loop inductance *L*.

The complex transfer function $v\left(t\right)/V_{g}$ is given by:

(18) $$\frac{v(t)}{V_{g}}=\frac{1}{1-jR\left(\frac{1}{wL(t)}-wC\right)},$$

and the magnitude of $v\left(t\right)/V_{g}$, i.e., $|v\left(t\right)/V_{g}|$ is:

(19) $$\frac{\left|v(t)\right|}{|V_{g}|}=\frac{1}{\sqrt{1+R^{2}{\left(\frac{1}{wL(t)}-wC\right)}^{2}}},$$

or equivalently,

(20) $$\left|v\left(t\right)|=\right|V_{g}|\frac{2\pi fL(t)}{\sqrt{R^{2}{\left(1-{\left(2\pi f\right)}^{2}L\left(t\right)C\right)}^{2}+{\left(2\pi fL(t)\right)}^{2}}}.$$

This output voltage amplitude could be approximated as a function of the inductance L(t) as follows:

(21) $$V=\left|v\left(t\right)\right|\approx \frac{1}{k_{1}L\left(t\right)+k_{2}},$$

and the amplitude change is given by:

(22) $$\Delta V=V_{v}-V_{nv},$$

$V_{v}$ being the voltage amplitude with vehicle presence, and $V_{nv}$ the voltage amplitude without that presence. Thus, we have:

(23) $$\begin{array}{cc} \Delta V & =V_{v}-V_{nv}=\frac{1}{k_{1}L_{v}\left(t\right)+k_{2}}-\frac{1}{k_{1}L_{nv}+k_{2}}=\frac{k_{1}\left(L_{nv}-L_{v}\left(t\right)\right)}{\left(k_{1}L_{v}\left(t\right)+k_{2}\right)\left(k_{1}L_{nv}+k_{2}\right)},\\ \frac{\Delta V}{V_{v}} & =\frac{k_{1}\left(L_{nv}-L_{v}\left(t\right)\right)}{k_{1}L_{nv}+k_{2}}=\frac{k_{1}\left(L_{nv}-L_{v}\left(t\right)\right)/L_{nv}}{k_{1}+\left(k_{2}/L_{nv}\right)}=\frac{k_{1}}{k_{1}+\left(k_{2}/L_{nv}\right)}S. \end{array}$$

Since $L_{nv}$ is constant, then the sensitivity *S* is:

(24) $$S=\frac{\Delta L}{L_{nv}}≅k\frac{\Delta V}{V_{v}},$$

i.e., the changes in the inductance of the inductive loop due to the presence of a vehicle modulate the amplitude of the fixed frequency carrier. In other words, the output voltage signal is *Amplitude Modulated* (AM) by the vehicle signature. Therefore, the demodulation of the AM waveform gives that vehicle signature and also, by means of an *Analogue-to-Digital Conversion* (ADC), the signature data. The bandwidth of the vehicle signature is mainly a function of the vehicle speed, the loop geometry, and the vehicle undercarriage features.

Let *n* be the bit number for ADC, and therefore $N=2^{n}$ the state counter. Let also $N_{t}$ be the count threshold, and then the threshold sensitivity is expressed as:

(25) $$S_{t}≅k\frac{N_{t}}{N}.$$

The problem of crosstalk for the amplitude ILDs with four or more loops [23] is solved by an RLC circuit per loop with the carrier frequencies of each loop spaced enough to include the signature bandwidth, and using a synchronous demodulator tuned to each carrier frequency.

## 3. Impact of Noise on Digital Detectors

In this section, we present a model to study the impact of noise on both resonant and amplitude detectors, which have been introduced in Section 2.

### 3.1. Impact of Noise on Resonant Detectors

**Figure 3:**
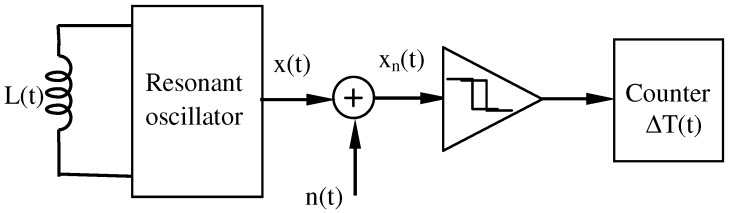
Block scheme of a resonant detector.

Figure 3 shows the block scheme of a resonant detector. Let L(t) be the equivalent inductance on the ends of the parallel resonant circuit constituted by this inductance and the equivalent capacity *C*. The oscillation frequency is given by Equation (15).

We will consider interferences caused by *Additive White Gaussian Noise* (AWGN), denoted as n(t), induced in the loop by ambient noise, like power lines, emissions from mobile phones, and so on. Therefore, at the comparator input we have:

(26) $$x_{n}\left(t\right)=x\left(t\right)+n\left(t\right),$$

where $x_{n}\left(t\right)$ is the signal plus noise. This signal must be converted, previously to be carried out to the counter input, to a digital pulse train. This conversion is performed by the comparator with hysteresis, which acts as a wave shaper. Then, the counter receives and counts the *m* counting cycles and measures the time interval ΔT, which provides the vehicle inductive signature.

### 3.2. Impact of Noise on Amplitude Detectors

**Figure 4:**
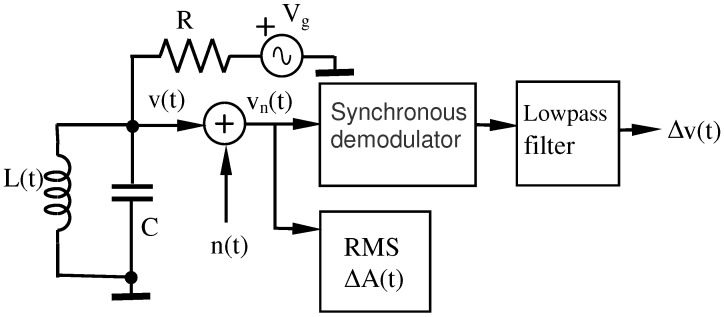
Block scheme of an amplitude detector.

Figure 4 shows the block scheme of an amplitude detector in presence of noise. Let v(t) be the signal at the output of the RLC circuit. Again, if we consider interferences produced by AWGN, denoted as n(t), the signal at the output of the RLC circuit is given by $v_{n}\left(t\right)$ as follows:

(27) $$v_{n}\left(t\right)=v\left(t\right)+n\left(t\right).$$

The *root mean square* (rms) value of this signal provides the inductive signature of amplitude ΔA(t).

#### Synchronous Demodulator (SD)

The signal modulated by the inductive signature s(t) can be written as:

(28) $$x\left(t\right)=\left(A+s(t)\right)\cos\left(wt\right),$$

and multiplying x(t) by the carrier, cos(wt), we have:

(29) $$y\left(t\right)=\left(A+s(t)\right)\;\cos^{2}\left(wt\right)=\frac{1}{2}\left(A+s(t)\right)+\frac{1}{2}\left(A+s(t)\right)\;\cos\left(2wt\right).$$

With a low-pass filter, we can eliminate the component of frequency 2w, so that, also removing the *Direct Current* (DC) component, the inductive signature Δv(t)=s(t)/2 is obtained (see Figure 4).

## 4. Proposed Design of an Inductive Detector

In this section, we present our implementation of the inductive signatures detector, referred to as SiVIDS. We will describe both hardware and software elements and the procedures for both measurement and registration of signatures. Our implementation has eight channels, allowing the registration of signatures of up to four lanes with dual loops in each lane or of up to eight lanes with simple loops in each lane. This covers most of the existing types of roads and makes the system easy to build thanks to the availability of a large number of standard integrated circuits with eight channels, like multiplexers, decoders, buffers, etc.

### 4.1. Colpitts Oscillator

**Figure 5:**
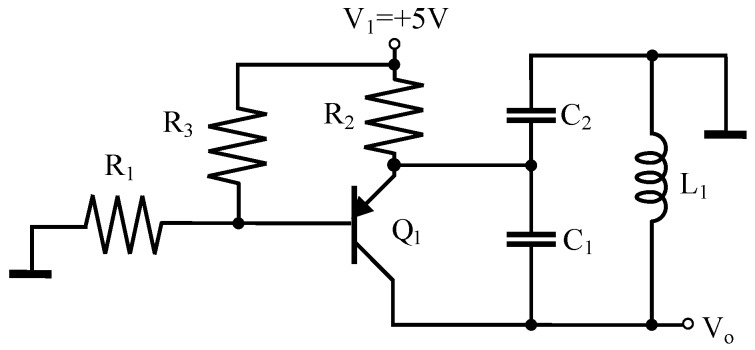
Circuit for the Colpitts oscillator.

The oscillation circuit employed in the proposed implementation is the well-known Colpitts oscillator, since it is the simplest resonant LC oscillator. Figure 5 shows the schematic of a Colpitts oscillator based on a pnp transistor in common base configuration connected to a tank circuit formed by the inductance $L_{1}$ of the inductive loop and the capacitors $C_{1}$ and $C_{2}$ that form the capacitive divider of the feedback loop [25,26].

The oscillation frequency is determined by the parallel resonant circuit formed by the inductance $L_{1}$, and the equivalent capacitor *C* obtained from the serial connection of $C_{1}$ and $C_{2}$, i.e.,

(30) $$f_{0}=\frac{1}{2\pi \sqrt{L_{1}C}},\;\text{with}\;C=\frac{C_{1}C_{2}}{C_{1}+C_{2}}.$$

### 4.2. Pulse Counter

**Figure 6:**
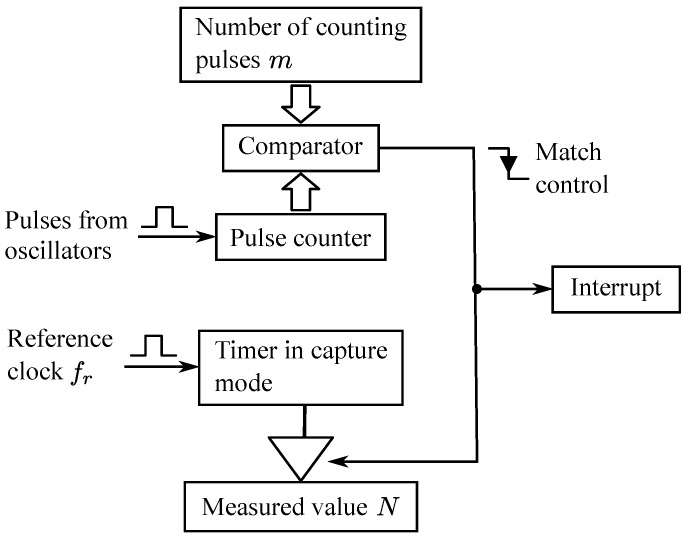
Measurement method by using comparison and capture.

Figure 6 shows a block diagram of the comparison and capture process necessary to measure the oscillation period automatically. The pulses from the oscillation loop that has been selected as input are carried to a counter input, so that when a fixed number of pulses *m* is reached, the measured value *N* is captured from a timer working at the frequency $f_{r}$ of the reference clock signal.

Since the basic measurement process is performed by hardware using interruptions, the delay time of interruption attention (known as *latency*) is not critical.

**Figure 7:**
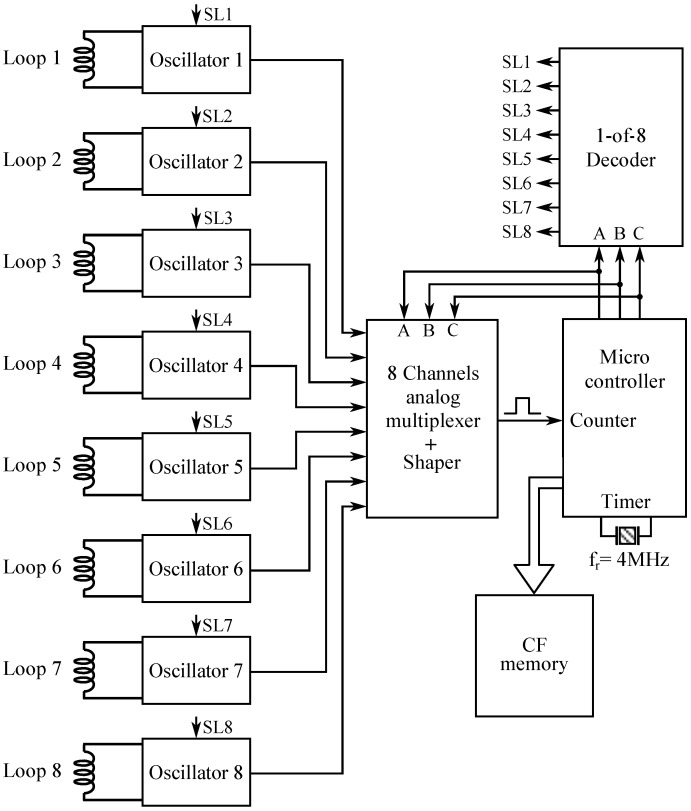
Multiplex system with eight inductive loops.

Figure 7 shows the practical implementation of the multiplex system with eight coils. It consists of eight Colpitts oscillators connected to eight inductive loops and an analogue multiplexer, which selects, at each instant, the oscillation signal of one of the loops using a decoder circuit. The use of eight oscillators instead of a single one allows us to avoid the introduction of an analogue multiplexer into the oscillation loop, which would be an additional error source.

The output signal of the multiplexer is carried to a shaping circuit, which converts the sinusoidal signal at its input into a digital pulse. That digital pulse is the input at the counter in the micro controller, which manages the entire system.

Due to the large amount of data captured by the system, a *Compact Flash* (CF) memory is employed for the recording of the signatures that will be subsequently analysed by a computer using signal processing algorithms. This off-line processing will allow us to perform vehicle classification and measurement of parameters such as speed or length, and even vehicle re-identification for monitoring and control applications of vehicular traffic.

**Figure 8:**
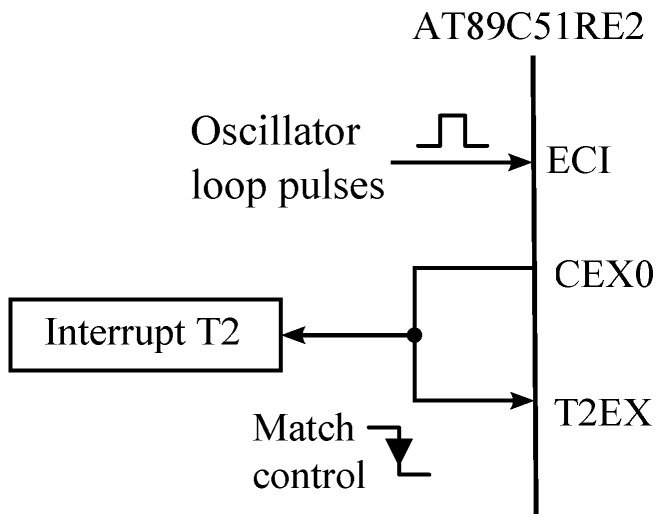
Interconnections for AT89C51RE2.

For the implementation of our system, we have chosen the AT89C51RE2 micro controller (Atmel, San Jose, CA, USA) since, firstly, it incorporates the comparison and capture unit needed in our application; and secondly, it can be easily interconnected to a CF memory bus. Figure 8 shows the interconnections for the AT89C51RE2 micro controller in our system. The output of the multiplexer is connected to the EC1 input, the CEX0 comparison output is carried to the T2EX input for capturing/interrupting, and the T2 timer is in capture mode.

**Figure 9:**
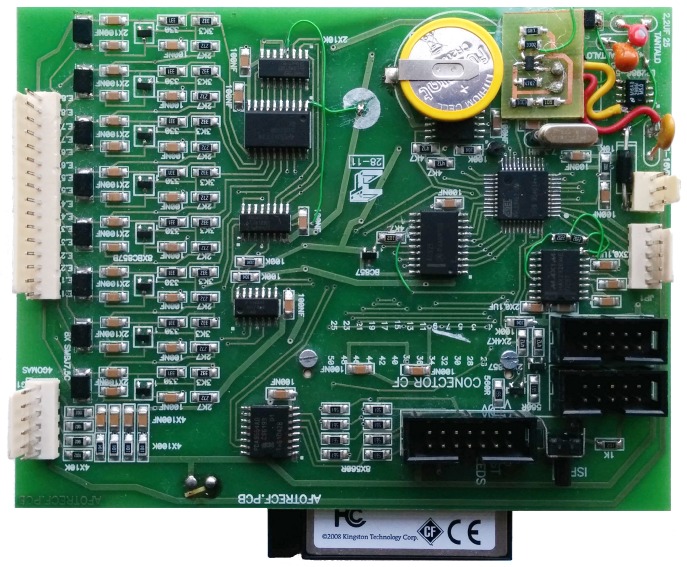
Photo of the hardware prototype.

Figure 9 shows a picture of the implemented hardware prototype. The left side of the board includes the eight oscillation circuits with multiplexing and a 16-pin connector for the connection of the eight inductive coils. The right side contains the micro controller and a *Real-Time Clock* (RTC) circuit with a lithium battery providing the date and time. The CF memory card used for the storage of the captured signatures can be seen at the bottom. One of the main advantages of the proposed system is that it can be implemented at a very low cost, thanks to its simplicity.

### 4.3. Measurement

As can be seen in Equation (12), the period of the oscillation signal in each loop is calculated as the number of cycles *N* of the reference clock signal in *m* cycles of the oscillation signal of that loop. The measurement of *N* is made by means of T2 interruptions generated by overflow (TF2) and by hardware automatic capture (T2EX). An initial number of oscillation cycles $m_{i}$ corresponding to the stability time of the oscillator start are discarded.

**Figure 10:**
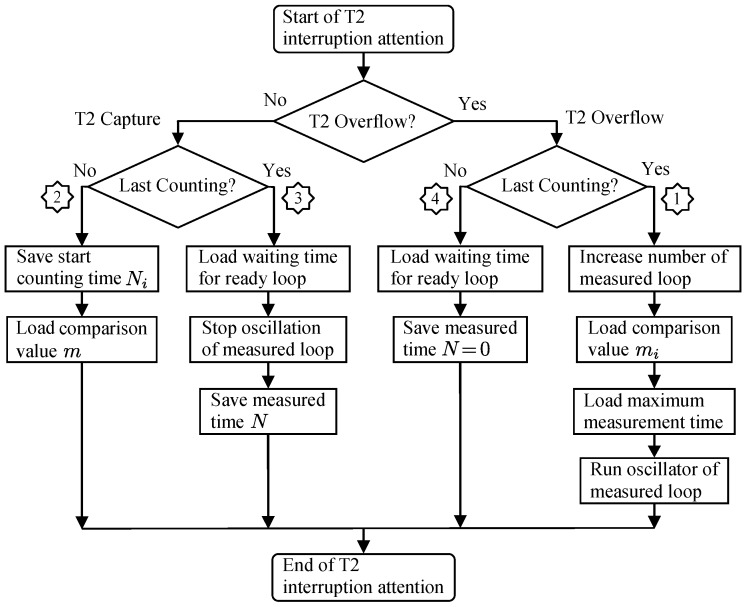
Flowchart of attention at T2 interruption.

Figure 10 shows a flowchart describing the process of the T2 interruptions’ attention. The measurement of each loop starts with the interruption by T2 overflow due to the delay time between loops required for the oscillation of the previous loop to completely disappear (the branch with number 1 in the figure). At that point, a new measurement loop is selected, the $m_{i}$ value is initialized to the number of initial start cycles, the maximum time for the measurement is established, and the corresponding oscillator is started.

Next, the branch marked with 2 in the figure is executed, so that the time $N_{i}$ at initial start cycles is measured and the number *m* of counting cycles to be measured is loaded.

Finally, the measurement process finishes with the interruption by T2 capture when *m* is reached, which corresponds to branch 3 in Figure 10. In this moment, the time interval between loops is loaded, and the oscillation loop stops and saves the measured time *N* obtained after subtracting the value $N_{i}$ of step 2.

Branch 4 only occurs in the unlikely event that the loop has problems with the start of the oscillation. In such a case, the inter-loop waiting time is loaded and *N* is set to zero, which indicates that the loop is not oscillating.

### 4.4. Registration

The oscillation period of the coils is continuously measured to determine the reference value of each coil at rest, i.e., without the presence of a vehicle. With the goal of adapting to the variations in the environmental conditions suffered by the coils, an adaptive algorithm, similar to the one described in [20], is employed. This algorithm tries to correct the reference value according to such external factors.

When the measured period of a coil is less than its reference value, which means that a vehicle is over the coil, the corresponding entry is made in the internal memory, storing the inductive signature of the vehicle.

**Figure 11:**
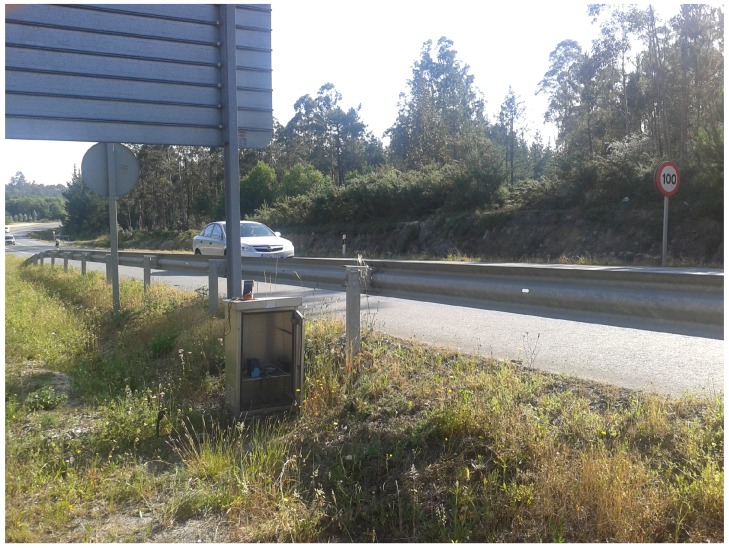
A photo of the measurement location, with GPS coordinates: 43.235941 (Lat.); −8.464462 (Long.).

**Figure 12:**
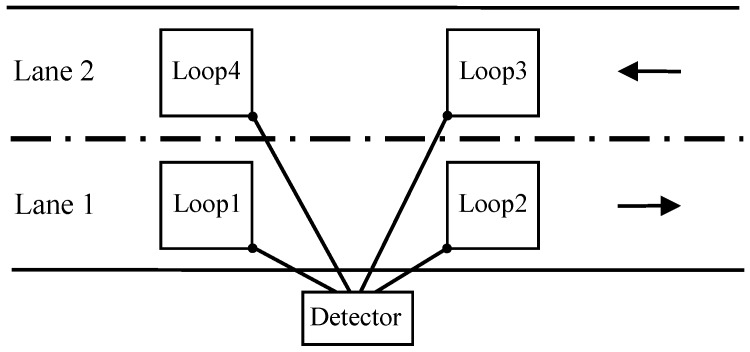
Interconnections from the inductive loop to the detector.

In order to test the hardware prototype we captured vehicle inductive signatures in Río Anllóns station in the AC-523 road (Ledoño-Meirama, Spain), kilometre 7. A picture of this location is shown in Figure 11. The detector equipment was located inside the cabinet of the Río Anllóns station, also shown in the photo. Since the road is two lanes wide (one for each direction), we have connected four inductive loop sensors, two on each side of the road. These sensors are squares with a side length of 2m and a distance between their centres of 5m (Figure 12).

## 5. Experimental Section

The inductive loop sensors work at a sampling period of T=10ms, or, equivalently, a sampling frequency of 100Hz. In any case, the sampling frequency could be easily increased by increasing the frequency of the reference clock, if required by other measurement conditions. Moreover, waiting times between coils are needed to guarantee that the oscillation vanishes in a coil before starting in the next one.

**Figure 13:**
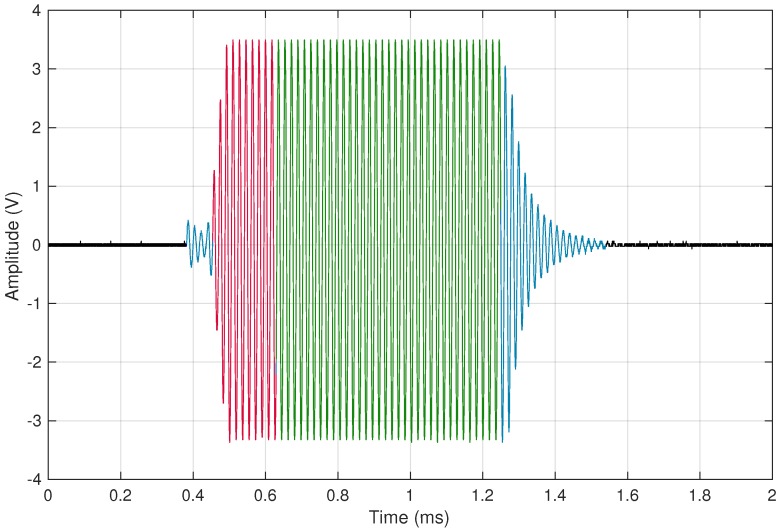
Capturing of a measurement of the coil oscillation.

Figure 13 illustrates the measured oscillation in a coil operating at a frequency of 56.2kHz, using $m_{i}=10$ initial cycles (in red) and m=35 measurement cycles (in green). The aforementioned waiting times at the beginning and at the end of the oscillations can also be observed (in blue) in the figure.

With the described sensors, two inductive signatures are obtained from each passing vehicle. These inductive signatures will be very similar, although there can be small differences due to the fact that they are taken in different loops and time instants, and, in general, also for different positions and accelerations of the vehicle. In order to display those signatures, we have developed a software tool using the development environment for a visual programming language Labview [27,28]. This tool allows us to download the file containing the signatures from the CF card. In addition to the ILDs, we placed a video camera for the recording of the passing vehicles, so we could associate each vehicle to its corresponding inductive signature.

It is important to note that we have acquired the real inductive signatures ΔT(t) using our SiVIDS prototype, and then from Equation (17), the equivalent inductance L(t) of each signature has been obtained. For the evaluation of the impact of the noise on resonant and amplitude detectors, AWGN noise will be added to the signals x(t) of Equation (16) and v(t) of Equation (18), respectively.

In our implementation, the Colpitts oscillator explained in Section 4.1 has $C_{1}=C_{2}=100\;nF$ and therefore C=50nF. Thus, an inductance of $L_{1}=100\;\mu H$ results in an oscillation frequency of 71.18kHz. The circuit has been designed for oscillation frequencies within the range [25kHz,100kHz] i.e., for coils with inductances between 50μH and 800μH. Therefore, it is not necessary to adjust the frequency of the LC oscillators (known as *tuning*).

## 6. Results and Discussion

In this section, we will show some results obtained from the inductive signatures captured using the prototype presented in this paper.

**Figure 14:**
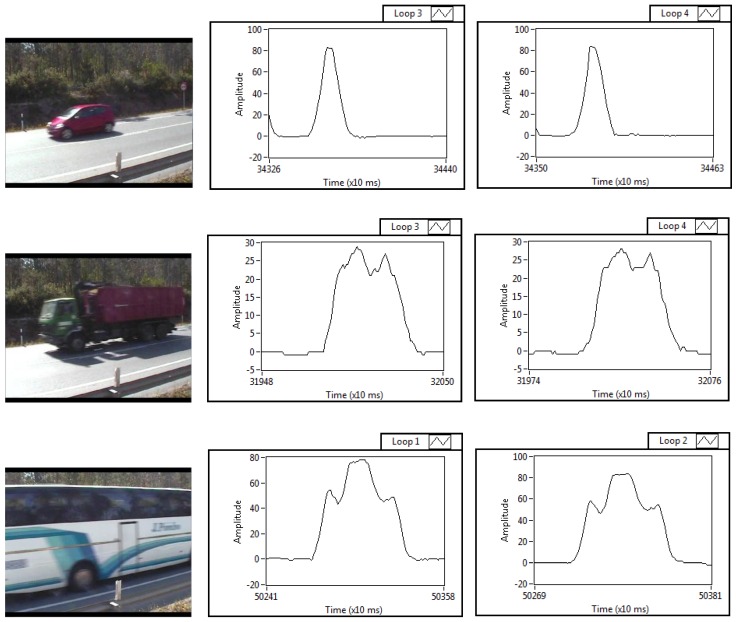
From left to right, the upper figures show the photo of a car and the respective signatures obtained with the Loops 3 and 4. The figures in the middle of the picture show a truck and its corresponding signatures captured also using the Loops 3 and 4. The lower figures display the photo of a bus and two signatures obtained with the first and the second loop, respectively.

Figure 14 shows the photos of three different vehicles and their corresponding inductive signatures obtained in the dual loops, as an example of the more than one thousand inductive signatures captured with our system. As it can be seen in the figure, there is a great similarity between the pair of signatures of any of the vehicles, in contrast with the significant difference in the signatures obtained for different types of vehicles. Thus, each type of vehicle (car, truck, bus...) can be classified under a unique inductive signature, which will depend on the parameters that define each of them, such as size, distribution of the metal mass, engine and axle location, spacing between the undercarriages and the road, etc.

### 6.1. Effect of Noise on Vehicle Inductive Signatures

**Figure 15:**
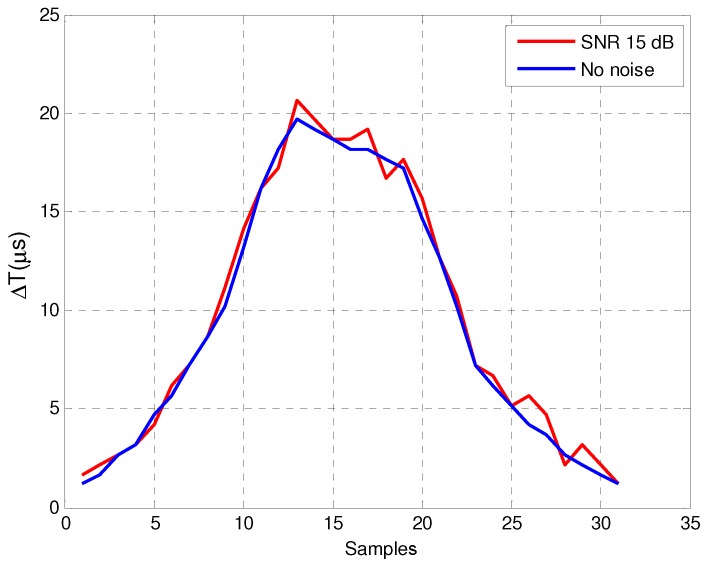
Inductive signature of the resonant detector with and without noise.

**Figure 16:**
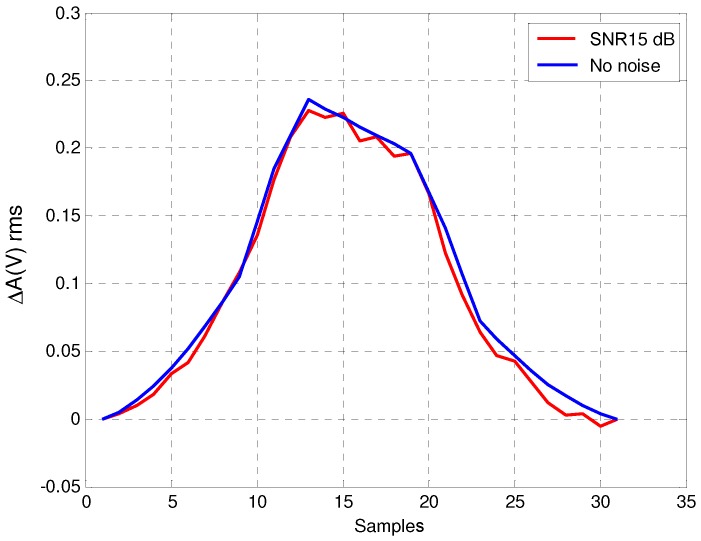
Inductive signature of the root mean square (rms) amplitude detector with and without noise.

**Figure 17:**
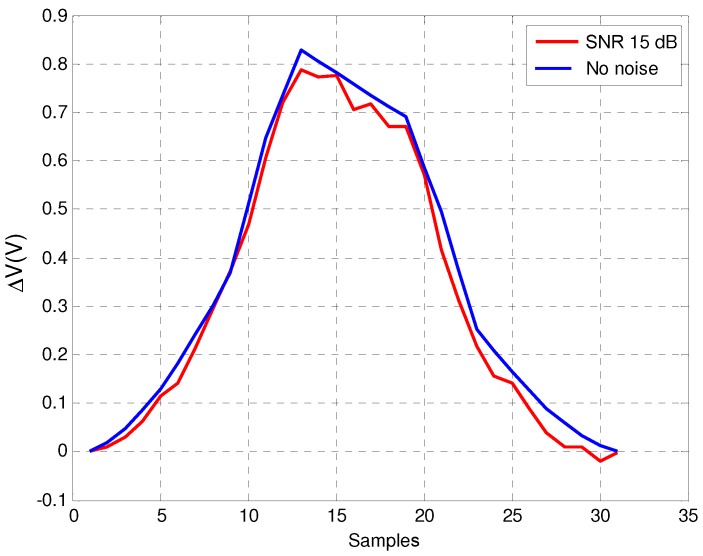
Inductive signature of the detector with synchronous demodulator with and without noise.

Figure 15 shows the real vehicle inductive signature obtained with noise—for a *Signal-to-Noise Ratio* (SNR) of 15dB—and with no presence of noise in the system. As we can see in the figure, even for 15dB of SNR, the shape of the noisy inductive signature is quite similar to that obtained with no noise at the detector input, which verifies that our resonant detector is robust against environmental noise. Figure 16 and Figure 17 show the impact of noise on the amplitude detectors for the same SNR.

Finally, we compare the performances of both resonant and amplitude detectors, in terms of SNR at the detector output. For this purpose, we calculate the output SNR as follows: firstly, we determine the level of signature signal without noise, i.e., $\sum \Delta x_{f}$; then, the level of noise is obtained as $\sum |\Delta x-\Delta x_{f}|$, where Δx is the signature signal plus noise; Finally, the output SNR is calculated as:

(31) $$\text{output}\;\text{SNR}=20\text{log}\frac{\sum \Delta x_{f}}{\sum |\Delta x-\Delta x_{f}|}.$$

**Figure 18:**
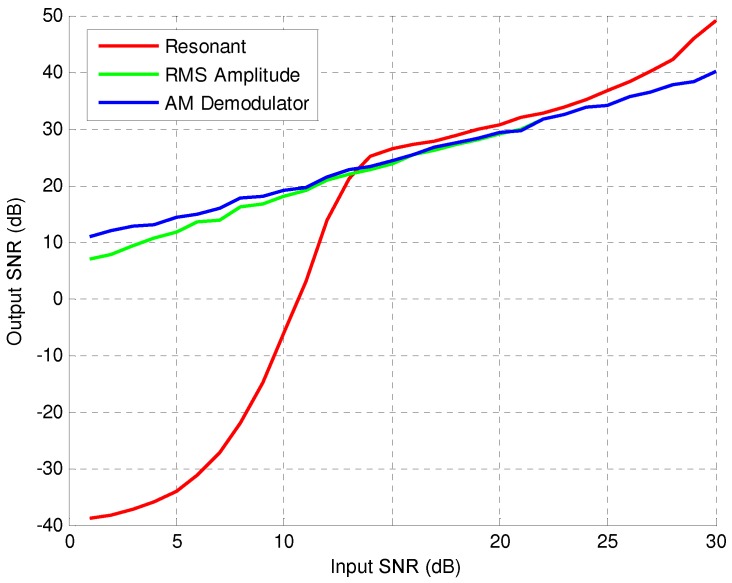
Average output Signal-to-Noise Ratio (SNR) for resonant and amplitude detectors.

The average output SNR is obtained by considering 556 real inductive signatures captured in the AC-523 road with the resonant detector, so that then the equivalent inductance L(t) of each signature is obtained. Figure 18 shows the average output SNR as a function of the input SNR for resonant and amplitude detectors. This figure shows a good behaviour against noise of the resonant detector for input SNR greater than 12dB, even better than that obtained for the amplitude detector. However, for SNRs lower than 12dB, the resonant detector is very sensitive to noise and, therefore, not useful for the purposes described in this work.

### 6.2. Effect of Noise on Speed Estimation and Vehicle Classification

In this subsection, we will evaluate the effect of the noise on two different applications usually required for ITS: speed estimation and vehicle classification.

**Figure 19:**
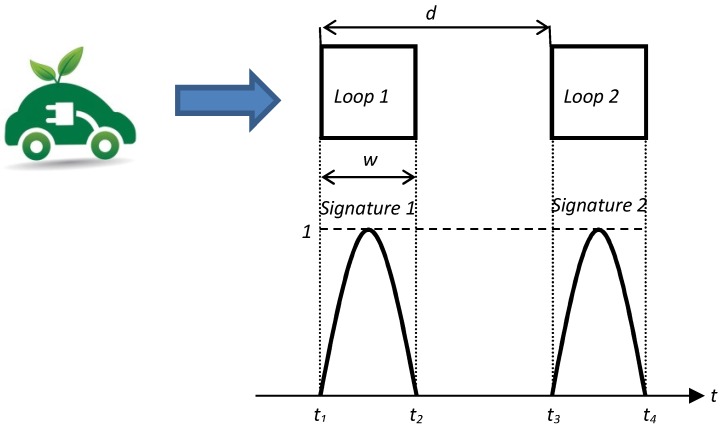
Scheme for time measurements.

According to Figure 19, the following time instants using double loop are determined:

- $t_{1}$: input time instant of the normalized vehicle signature 1;
- $t_{2}$: output time instant of the normalized vehicle signature 1;
- $t_{3}$: input time instant of the normalized vehicle signature 2;
- $t_{4}$: output time instant of the normalized vehicle signature 2.

The standard method for speed estimation uses the following expression, according to the aforementioned notation [29,30],

(32) $$\hat{s}=\frac{1}{2}\left(\frac{d}{t_{3}-t_{1}}+\frac{d}{t_{4}-t_{2}}\right),$$

where *d* is the distance between loop centres. From this expression, the vehicle length can be directly obtained using the following estimator:

(33) $$\hat{L}=\hat{s}\times \frac{\left(t_{2}-t_{1}\right)+\left(t_{4}-t_{3}\right)}{2}-w,$$

with square loops of side length *w*. For vehicle classification, the vehicles passing on the road will be classified using a threshold-based criterion as indicated in Table 1.

Notice that $\epsilon_{1}$ and $\epsilon_{2}$ are the thresholds empirically obtained from a training stage. For this training, only the Loops 1 and 2 are used. The value corresponding to the threshold $\epsilon_{1}$ was obtained by maximizing the success rate in vehicle classification when only small and medium vehicles are considered, while the threshold value $\epsilon_{2}$ is the result of a similar maximization when only medium and large vehicles are computed. The optimum values for those thresholds are $\epsilon_{1}=5.6\;m$ and $\epsilon_{2}=6.5\;m$.

**Figure 20:**
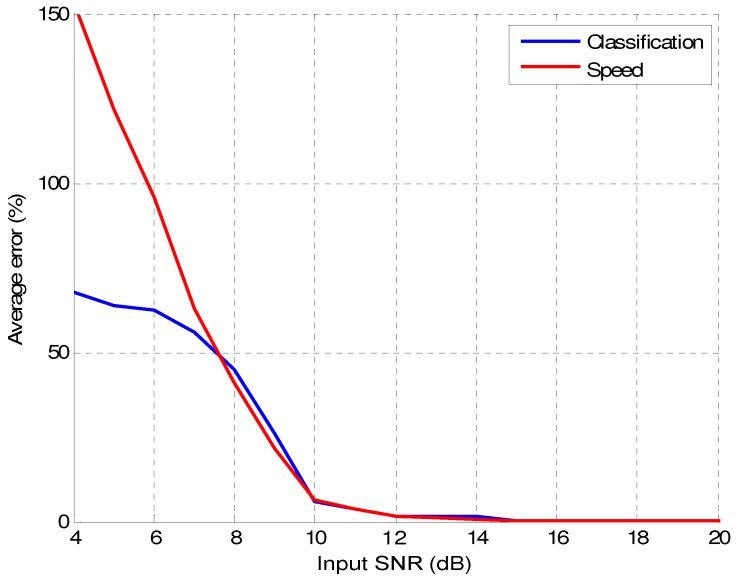
Influence of Additive White Gaussian Noise (AWGN) on vehicle classification and speed estimation errors.

Applying these methods, Figure 20 shows the influence of AWGN on vehicle classification with the length-based criterion and on vehicle speed estimation. The error percentage for vehicle classification is calculated as follows:

(34) $${\text{error}}_{c}\left(\%\right)=100\times \frac{c_{\mathrm{AWGN}}}{c_{0}},$$

where $c_{\text{AWGN}}$ is the total number of misclassified vehicles under the presence of AWGN, calculated with respect to the classification without noise, and $c_{0}$ is the total number of vehicles. On the other hand, the percentage of error for speed estimation is calculated as follows:

(35) $${\text{error}}_{s}\left(\%\right)=100\times \frac{|s_{\text{AWGN}}-s_{0}|}{s_{0}},$$

where $s_{\text{AWGN}}$ and $s_{0}$ are, respectively, the estimated vehicle speeds with and without the presence of AWGN. All the results have been averaged for the dataset collected from the AC-523 road.

This figure allows us to conclude that in both applications the effect of noise is almost negligible for SNR above 12dB.

## 7. Conclusions

Experimental results have shown that ILD sensitivity is extremely repeatable for fixed sizes and geometries of both the loop and the vehicle, and for a fixed distance between them. Therefore, we have shown that the sensitivity can be approximated by variations of period and amplitude of voltage of the oscillation signal. Moreover, resonant ILDs show an adequate compromise between reliability and cost, which determines that such detectors have been selected for our practical implementation.

In this paper, we have presented a simple module for the capture of inductive vehicle signatures based on TDM. The implemented system performs a sequential scanning using analogue multiplexing of up to eight oscillators and detects the presence of a vehicle by means of a shift in the period of the signals from the selected oscillator. It subsequently captures the inductive signature of the detected vehicle by measuring the time it needs to count a fixed number of pulses.

In the experimental results obtained from measurements in a real scenario using dual loops, we observed a good similarity between the pair of signatures obtained from the same vehicle and a significant difference between the signatures corresponding to different vehicles, which validates the good performance of our implementation and enables its use in applications such as vehicle classification, speed and length measurement using only one loop, and re-identification of vehicles for supervision and control tasks in vehicular traffic.

Moreover, the performance of the resonant detector proposed in this work is validated in the presence of AWGN determining an input SNR higher than 12 dB.

## Figures and Tables

**Table 1:** Vehicle classifiation using a length-based criterion, i.e., based on the $\hat{L}$ estimator of Equation (33).

Vehicle Classification	Small	Medium	Large
Type of vehicles	Car	Large car, van	Truck, bus, trailer
Number of vehicles (manually pre-classified with a video camera)	680	61	168
Decision rule	$\hat{L}\leq \epsilon_{1}$	$\epsilon_{1}<\hat{L}\leq \epsilon_{2}$	$\hat{L}>\epsilon_{2}$
